# Targeted Anterior Cervical Epidural Blood Patch in a Patient with Spontaneous Intracranial Hypotension

**DOI:** 10.1155/2022/8872775

**Published:** 2022-09-19

**Authors:** Ravindra Singh Shekhawat, Ming Hui Yong, Si Ying Julienne Keong, Kunihiko Chen, Chow Wei Too, Shahul Hameed

**Affiliations:** ^1^Department of Neurology, National Neuroscience Institute-Singapore General Hospital, Singapore; ^2^DUKE-NUS Medical School, Singapore; ^3^Department of Vascular and Interventional Radiology, Singapore General Hospital, Singapore

## Abstract

A 45-year-old male was admitted with severe orthostatic headache secondary to spontaneous intracranial hypotension. He had the site of cerebrospinal fluid (CSF) leakage identified at the anterolateral aspect of the C7-T1 spinal level. He first underwent a conventional posterior-approach cervical epidural blood patch (EBP) which provided immediate relief to the patient's symptoms; however, his symptoms recurred two days later. To better target the anterolateral leakage site, we employed an anterior-approach EBP under computed tomography (CT) guidance. After this attempt, the patient experienced complete relief of his symptoms, and the headache eventually resolved.

## 1. Background

Spontaneous intracranial hypotension (SIH) is a condition first described in 1938. SIH often occurs secondary to cerebrospinal fluid (CSF) leakage at the spinal level. Symptoms of SIH include orthostatic headaches, which can be associated with other symptoms including nausea, vomiting, photophobia, cranial nerve palsies, visual blurring, tinnitus, and neck stiffness [1]. On magnetic resonance imaging (MRI) of the brain, pachymeningeal enhancement, subdural hygroma, brain sagging with cerebellar tonsillar descent, pituitary enlargement, or venous sinus dilation is commonly visualized [1–3]. MRI of the spine, computed tomography (CT) or MRI myelogram, and radionuclide cisternography can also help to identify the site of leakage [3]. Treatment of SIH can be conservative with bed rest, hydration, caffeine, and analgesics. If symptoms persist or are severe, epidural blood patches (EBPs) are another treatment option. We present a case of a middle-aged male diagnosed with spontaneous intracranial hypotension, with an identified site of CSF leakage at the anterolateral aspect of the C7-T1 spinal level, who had no or minimal relief from conventional posterior-approach cervical EBP. He eventually underwent a more targeted anterior-approach cervical EBP approach. Our case demonstrates the feasibility and efficacy of an anterior-approach cervical EBP for SIH patients whose sites of CSF leak are anteriorly located.

## 2. Case Presentation

A 45-year-old Indian gentleman with a past medical history of hyperlipidemia and migraine presented to an inpatient Neurology ward of a tertiary hospital, complaining of a one-month history of headache that had begun after cervical manipulation during a neck massage. He complained of pain over the occipital region and over the vertex which radiated down the neck. This pain was shooting in nature at the occipital region and pressing in nature at the vertex. The onset was not thunderclap in nature. Most significantly, the pain had an orthostatic quality, worsening in intensity within a few minutes of sitting or standing and improving within a few minutes of lying down. Otherwise, the patient did not experience nausea, vomiting, limb weakness or numbness, or other focal neurological symptoms. He had no fever, photophobia, nor phonophobia. He had been treated previously with various analgesics which did not provide any relief. His last migraine headache had occurred more than ten years ago and differed in character from his current headache. A full neurological examination did not demonstrate any focal neurological deficits.

### 2.1. Investigations

**Figure 1:**
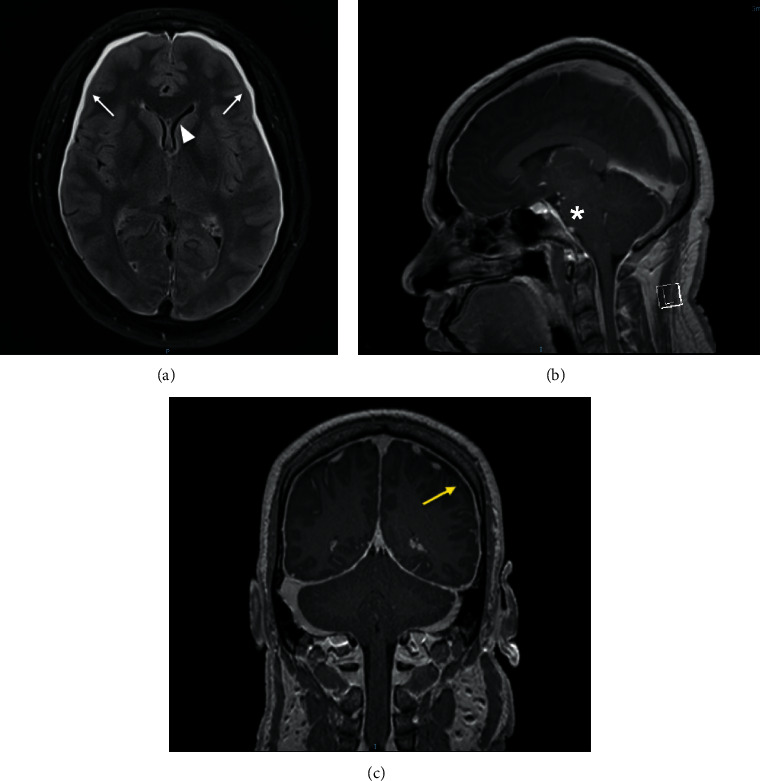
MRI of the brain showing features of intracranial hypotension. (a) FLAIR: bilateral subdural hygromas (white arrows) and slit-like ventricle (white arrowhead). (b, c) Post-contrast: reduced pre-pontine space (asterisk) and diffuse pachymeningeal enhancement (yellow arrow).

**Figure 2:**
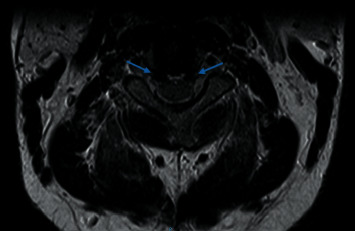
Contrast-enhanced MRI of the cervical spine, axial view. T2-weighted image: prominent epidural veins (blue arrows).

The patient underwent extensive investigations to confirm the diagnosis of SIH and the site of the CSF leak. Contrast-enhanced MRI of the brain showed typical features of SIH (Figures 1(a)–1(c)): diffuse pachymeningeal enhancement, distension of the cerebral venous sinuses, bilateral subdural hygromas, reduced pre-pontine space, and slit-like ventricles. Contrast-enhanced MRI of the cervical spine showed supporting imaging features of intracranial hypotension, including a small thecal sac and prominent epidural veins in the cervical spine (Figure 2); no epidural CSF fluid collection was visualized.

**Figure 3:**
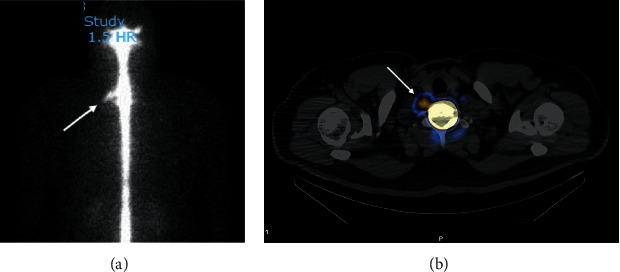
(a) Radionuclide (Tc-99 DTPA) cisternogram at 3-hour interval showed Tc-99 DTPA tracking out at the right of the upper cervical/thoracic vertebrae (white arrow). (b) SPECT at 2-hour time interval, axial view, showed focal extrathecal tracer accumulating at the right anterolateral aspect of the C7/T1 level (white arrow).

To localize the CSF leak, the patient underwent radionuclide cisternography, which was more readily available in our institution compared to CT or MRI myelography. Intrathecal injection of technetium (Tc-99m) diethylenetriamine pentaacetic acid (DTPA) was administered, with serial static planar images acquired at 1.5-hour, 2-hour, and 3-hour intervals as per our institute's protocol. Within this time, the site of CSF leak was identified (and the study did not have to be prolonged up to 24 hours): his cisternogram showed tracer tracking out to the right of the upper cervical/lower thoracic vertebrae as early as 1.5 hours (Figure 3(a)). Supplementary single-photon emission computed tomography (SPECT) of the cervical and thoracic spine at the 2-hour interval showed focal extrathecal tracer accumulation at the right anterolateral aspect of the C7/T1 level, likely representing CSF tracking out from the right C7/T1 neural foramen (Figure 3(b)).

### 2.2. Treatment

**Figure 4:**
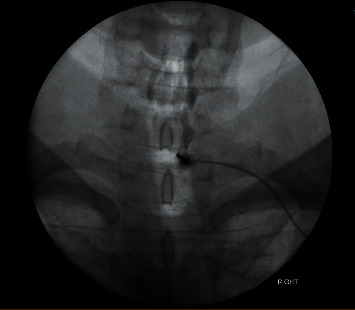
Posterior-approach cervical epidural blood patch under fluoroscopy.

Conventional treatment with bed rest, intravenous hydration, analgesia, and encouraged caffeine intake was initiated; however, this provided only minimal pain relief for the patient. Subsequently, the patient underwent a conventional posterior-approach cervical EBP performed by an experienced anaesthetist. At the right paramedian cervical region, 25 mL of autologous blood was injected into the epidural space at the C7/T1 level of the spine from the posterior approach, under fluoroscopy guidance (Figure 4). He was not on aspirin or anticoagulant therapy.

Following the procedure, the patient reported improvement of the headache by the next day and complete resolution of symptoms upon discharge. However, his headache later recurred 2 days after the initial EBP procedure and continued to increase in severity. He was re-hospitalized 12 days after the EBP. A CT-brain performed showed persistent slit-like ventricles and increased size of bilateral subdural collections with iso-to-hyperdensity suggestive of interval bleeding and interval worsening of his SIH. At this point in time, the patient was extremely distressed by his symptoms—in addition to being severe and prolonged, they limited his activities greatly. While he agreed to a repeat EBP attempt, he found difficulty in accepting the possibility of yet another relapse after requiring further epidural blood patch attempts or even consideration of surgical intervention should his symptoms remain refractory to EBPs.

**Figure 5:**
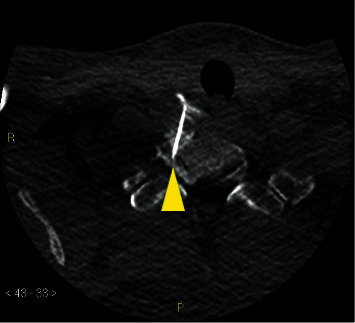
CT-guided injection. Axial section showing the tip of the 22G spinal needle at the right C7/T1 foramen. The contrast was injected to confirm flow centrally (yellow arrowhead).

The patient underwent a second cervical EBP 20 days after the first EBP, which was now CT-guided and performed by an interventional radiologist who used an anterior approach to better target the right anterolateral site of the C7/T1 CSF leakage. The choice of the anterior transthyroid approach afforded the greatest precision to target the CSF leak site, and a 22G spinal needle was inserted via the transforaminal route followed by injection of a total of 7 mL of autologous blood around the C7/T1 epidural space under CT guidance (Figure 5).

### 2.3. Outcome and Follow-Up

The patient tolerated the procedure well with no neurological deficits or bleeding complications post-procedure. The patient experienced immediate and sustained improvement in his headache after the second EBP, and an interval MRI brain performed three weeks later showed partial resolution of the SIH changes. One month after the second EBP, the patient reported complete resolution of the headache and has returned to work since. More than a year after the targeted EBP procedure, the patient reported no headache recurrence, reflecting the successful outcome of this procedure.

## 3. Discussion

In this case report, we report a patient with SIH due to CSF leak after neck manipulation who needed two EBPs, the second being an anterior approach prior to of resolution of symptoms. The suspicion of SIH arises in patients with headaches that are exacerbated by upright posture and improved in the supine position [1]. Contrary to its name, SIH does not always occur spontaneously and may be classified as either primary SIH where no attributable cause is found or secondary SIH due to a provocative cause, such as spinal trauma, diagnostic/therapeutic spinal procedures like lumbar puncture, chiropractic manipulation, and degenerative spine disease [2]. The etiology of a spinal CSF leak can also be classified as resulting from a dural tear (type 1), meningeal diverticulum (type 2), or CSF-venous fistula (type 3) [4]. Our patient likely developed SIH from a dural tear following chiropractic manipulation of the cervical spine.

MRI brain generally has radiological features of low CSF pressure such as tonsillar herniation, flattening of the pons along the clivus, and displacement of the optic chiasm as primary features of SIH. It is postulated that the pachymeningeal enhancement and subdural effusion reported in few cases of SIH are secondary features that are due to dilated leaky meningeal vessels and rupture of bridging veins, respectively, which are thought to be due to the downward displacement of the brain due to low CSF pressure [3, 5, 6]. In SIH, CSF examination may be unremarkable or show mild pleocytosis (due to meningeal hyperemia associated with movement of cells into the subarachnoid space [7]) or mildly raised protein.

In patients with SIH, the lower cervical spine and upper thoracic spine are the most common sites of CSF leak [3]. Once SIH is suspected, imaging modalities such as CT/MRI myelography and radionuclide cisternography are often used to identify the site of CSF leak [3]. The characteristic finding is paradural extravasation of the radioisotope, as was observed in our patient. In some cases, often a slow cephalad extension of the radioisotope is noted, which indirectly signifies volume loss of CSF [8].

SIH can be conservatively managed by bed rest, analgesia, increased fluid intake, increased caffeine, or increased salt uptake. If conservative treatment fails, an EBP can be offered. EBP is thought to be effective in stopping the orthostatic headaches by the injected autologous blood in the epidural space forming a gelatinous seal at the site of leak and by increasing the intrathecal pressure which in turn can relieve the symptoms of CSF hypovolemia [9, 10].

For our patient with a clear right C7/T1 CSF leak, we had to decide between treating him upfront with a “blind” EBP or with an EBP targeted at the CSF leak site. Conventionally, “blind” EBP at the lumbar region was the first choice for patients who failed conservative treatment; however, lumbar EBPs may not be theoretically as effective if the site of leak is at the cervical or thoracic region. While no randomised controlled trials exist, observational and case series data suggest that targeted EBPs are more efficacious than blind EBPs [9, 11–13]. However, cervical epidural blood patch for our patient would be associated with increased risks, namely, injuries to the nerve roots or spinal cord, the possibility of blood and chemical meningitis, and the risk of injury to critical structures such as the carotid vessels, trachea, and esophagus [10, 14]. Despite these risks, given our patient's distress from severe symptoms, the presence of subdural collections with possibility of progression to developing worsening subdural hematoma and mass effect, and our concerns that a lumbar EBP would not be successful, we first attempted a targeted cervical EBP with a posterior approach by an experienced operator. However, while he had some relief, the relief was not sustained. Given the anterior position of his leak, the second cervical EBP was performed from an anterior approach with CT guidance, with a lower volume of autologous blood injected to minimize the risk of complications, and the procedure was successful in alleviating his symptoms without any side effects.

While targeted EBPs are common in established centers, they are usually from a posterior approach. Reports of anterior-approach cervical EBP for an anteriorly located cervical CSF leak are scarce. Park and Villblanca were the first to report an anterior-approach cervical EBP under fluoroscopic guidance for their patient with refractory severe SIH who had failed 2 prior lumbar EBPs in 2013 [14]. For our patient, CT rather than fluoroscopic guidance was utilized to allow better EBP placement at the exact site of CSF leak visualized on his previous SPECT-CT study and to minimize risk to other surrounding structures such as the thyroid gland that lay in the path of the spinal needle's planned approach. CT guidance can be considered in such cases where visualization of the neck structures is preferred or where a very focal location of the CSF leak to be targeted has been identified in prior CT or MRI spine imaging.

## 4. Conclusion

To the best of our knowledge, this is one of the few reports in the literature describing the successful use of an anterior-approach cervical epidural blood patch to treat a case of refractory SIH. Our report adds to the literature our experience and supports the feasibility of anterior-approach cervical EBP for effective treatment of SIH due to anterior cervical CSF spinal leaks.

## Data Availability

Due to Singapore's personal data protection act, no further supporting data can be provided.
